# Narratives of Women and Gender Relations in Chinese COVID-19 Frontline Reports in 2020

**DOI:** 10.3390/ijerph20054359

**Published:** 2023-02-28

**Authors:** Shuoyu Fang, Li Zou

**Affiliations:** School of Foreign Languages, Shanghai Jiao Tong University, Shanghai 200240, China

**Keywords:** gender relations, news discourse, COVID-19, Chinese newspaper, appraisal analysis

## Abstract

This article analyzes the representation of women in Chinese news reports about COVID-19 in order to examine the consequences of the pandemic on gender relations in China. It draws on the linguistic framework of appraisal theory for identifying evaluative language and takes Chinese news reports on the COVID-19 frontline in 2020 as the major data sources. The study finds that while the narrative about women’s capacity in combating the virus, resolution in the face of adversity, and sense of responsibility help build a shared feeling of community to reconstruct the disturbed social order, the descriptions about the evaluation and feelings of female characters lead to undesirable outcomes in gender relations in China. Specifically, the newspapers’ reports on COVID-19 mainly focus on group interests and accomplishments and overlook women’s contributions in containing the pandemic. Meanwhile, the news reports devoted to constructing model female characters that highlight transcendent qualities place considerable pressure on everyday women. Furthermore, journalists tend to infuse their reports with gender bias when depicting women, including aesthetic appreciation of appearance and a focus on emotional reactions and domestic roles, which hinders the professional identity of women. This article sheds light on gender relations in China amid the pandemic, as well as the study of gender equality in media discourse.

## 1. Introduction

The COVID-19 pandemic has brought gender concerns to the forefront of the general public, with a special emphasis on the impact that job losses and lockdowns have had on women’s livelihoods. Worldwide national and international organizations, represented by the National Bureau of Economic Research in America, the United Nations Sustainable Development Group, the International Monetary Fund, and the Institute of Labour Economics, have released several policy briefs or working papers discussing the impacts of COVID-19 on gender equality [1,2,3,4]. Research that addresses gender problems during COVID-19 has been conducted in a sizable number of nations worldwide, including the United States, Israel, Argentina, Spain, Italy, Japan, and countries across Africa [5,6,7,8,9,10,11,12,13]. They conduct survey-based research and touch upon women’s psychological well-being [7,11,13], domestic or childcare responsibilities [8,9,12], daycare and eldercare [7,13], and work-related outcomes [5,6,9,10,11]. Gender differences exist regarding the employment rate, division of domestic work, and changes in purchasing behaviors, as well as changes in income and earnings.

Notably, concern for women’s well-being underscores their pivotal roles in combating the pandemic. Magar et al. [14] and Bonio et al. [15] investigated the female healthcare workforce in hundreds of countries and discovered that the female share of employment is around 70% in the health and social sectors, with nursing and midwifery being the most female-dominated occupations. In addition, a policy note by the World Bank Group on gender dimensions of the COVID-19 pandemic [16] shows that women make up 88% of workers in personal care, 69% of health professionals, and 60% of workers in food preparation. In China, female healthcare workers consisted of two-thirds of the total of 42,000 medical staff that were sent to Hubei Province to fight against the pandemic, as reported in a news conference held by the National Health Commission of China on Women’s Day in 2020 [17]. Moreover, in a news conference held by the State Council’s Joint Prevention and Control Mechanism Comprehensive Team of China in 2020, it is reported that female nurses accounted for 90% of all the nurses sent to support Hubei [18]. The occupational segregation by gender unveils the underlying gender inequalities in labor markets. It is important to analyze how women have been socially represented in the 2020 COVID-19 pandemic and whether Chinese women’s roles have been well recognized during this time.

This article uses appraisal-based discourse analysis to analyze the impact of narratives in Chinese COVID-19 frontline reports upon gender relations, focusing on examining the gendered evaluative language targeted at men and women. Appraisal is a technical term in systemic functional linguistic theory for the system describing semiotic resources that realize stances or attitudes. It is concerned with the subjective presence of writers/speakers in texts as they positively or negatively evaluate both the material they present and those with whom they communicate. The writers/speakers in texts construct communities of “shared emotions, tastes, and normative assessments” [19]. In news reporting (including Chinese COVID-19 frontline reports), the ways journalists represent reality show their stances towards people, things, and events, as well as gender relations. By means of the appraisal system, we can “capture the delicate interweaving of explicit and implicit attitudes that powerfully positions readers of narratives” [20]. With variable linguistic mechanisms for analyzing different degrees and types of evaluation, the appraisal system is thus a useful tool to investigate the impact of Chinese news reports on COVID-19 upon gender equality.

The rest of the article is structured as follows. Section 2 details the criteria underpinning the data collection and introduces the appraisal framework for the analysis of evaluative meaning in language. Section 3 overviews the distribution of attitudinal resources, with detailed exemplification of typical patterns. Section 4 summarizes the gendered attitudinal representations and their implications for gender relations in China, and the underlying socio-cultural values are also discussed.

## 2. Materials and Methods

The data for this study are extracted from a specialized website for new coronavirus pneumonia prevention run by Wuhan University [21]. The website complements the medical and scientific expertise provided by one of the flagship universities in Wuhan to manage the pandemic. The news trends in Chinese society during the pandemic are well-represented on this website regarding the timely and authoritative source of information and its function as a reliable channel for public health communication. It has a systemic design of columns, containing News Focus, Work News, COVID-19 Pandemic Briefing, Notice and Announcement, Donation Information, Prevention and Treatment, Convenience Service, and Counter-rumors. Taking into account the development of COVID-19 in Wuhan, where an absolute lockdown came into force on 23 January 2020 and was lifted on 8 April 2020, we mainly focus on news reporting in January, February, and March. The News Focus category comprised 105 pages of news for that period, and each page contained 15 articles. Based on the alphabetical order of 105 pages, a systematic sampling method was deployed to select every 5th page. This produced a sample of 21 pages, which contained 315 articles. Of these, news referring to policies, reviews, conferences, and theories with no protagonists or spokespersons are of little use to our research aim, so these were removed. In total, 150 pieces of news were collected, of which 23 reports focus on female frontline nurses and caregivers on International Women’s Day.

Table 1 lists the agency information of the data with links, text numbers, and brief introductions to each media organization. As shown in the introduction of Table 1, these media cover large newspaper groups in China, such as *People’s Daily* and Xinhua News Agency, specialized newspapers focusing on health, science, and theory, and leading daily newspapers based in different provinces of China. Thus, these statistics encompass major media outlets on both a national and provincial level, making them a good indicator of public opinion on either a local or national level. Most of the news media have been revamped with e-papers and news apps. Among all the news publishers, Xinhua has a more complete release system featuring multiple channels and covering all kinds of administrative levels, such as *Xinhua Daily Telegraph* and xhby.net in our data. Similarly, China News Service has its subordinate *China Newsweek* magazine, and *People’s Daily* runs an online forum. As for the audience size, national media is overwhelmingly more widely read than specialized newspapers and provincial media. The reason is that the news media at the national level hold predominance in communication in response to breaking national news. They keep in alignment with the central guidance of the government. By contrast, provincial media are oriented towards the guidelines from the central government and produce localized news reports, e.g., reporting the rescue medical teams dispatched from their provinces amid the pandemic.

In terms of the evolution of the agendas set by these media organizations, reports in January and March have different focuses. When the Chinese authority identified and declared the coronavirus outbreak as an epidemic emergency in January 2020, the overwhelming majority of the news comprised reports about the latest cases and deaths of COVID-19, as well as government instructions and work plans for virus control. After the situation was potentially managed in March, more journalists were on the scene personally to see what was happening in the hospitals and to report on the challenges faced by healthcare workers and patients. Our focus in this study is the examination of how men and women were depicted via evaluative language during this second phase.

**Figure 1:**
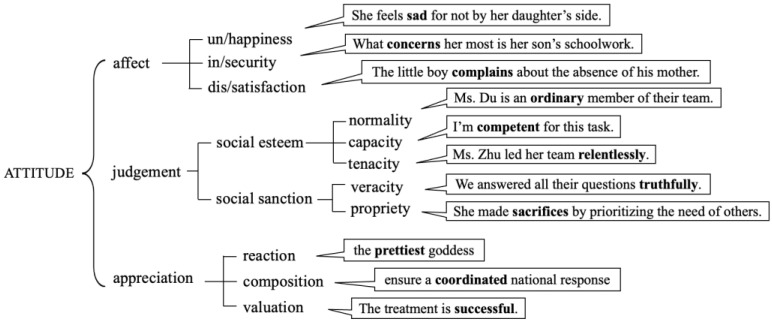
Basic System of attitude.

As attitudes permeate all levels of linguistic description and are always active in news discourse, female narratives and gender relations can be explored from the evaluative words and expressions in Chinese newspapers. We draw on the appraisal model from Systemic Functional Linguistics, which is regarded as “the most fully theorized and elaborated account of evaluation at present” [20] in linguistics. It has been developed by grammarians as a discourse semantic system that motivates a lexis-oriented classification and helps manage the analysis of evaluation in discourse [19]. It allows us to capture emotional, ethical, and aesthetic aspects of evaluation (attitude); it makes space for the adjustment of amplitude and the precision of attitudes (graduation); and it enables us to investigate the play of voices within and across texts (engagement) [20,22]. This paper will focus on the subsystem attitude, which incorporates affect (people’s feelings), judgement (people’s character), and appreciation (the value of things). The system is shown in Figure 1, with examples on the right of each feature drawn from the data (appraisal words in bold).

affect is the set of choices to do with ‘emotional responses’—expressing reactions to and feelings about things, such as liking or fearing [20]. This is based on Martin’s observations of his young son’s evolving engagement with the world [22]. It groups emotions into un/happiness, in/security, and dis/satisfaction. Un/happiness is concerned with emotional states, such as sadness, hatred, or love; in/security covers feelings of safety or threat in relation to our environment, such as anxiousness or confidence; dis/satisfaction deals with our feelings of achievement and frustration, such as satisfaction or boredom.

The expression of personal reactions via affect develops as we are socialized into a culture and into cultural institutions. These feelings become institutionalized as ethics or morality, forming the judgement system, and as aesthetics or value, forming the appreciation system [23]. judgement differs between personal judgements of admiration or criticism (social esteem) and moral judgements of praise or condemnation (social sanction). The former has to do with how unusual (normality), how capable (capacity), or how resolute (tenacity) someone is. The latter has to do with how truthful (veracity) or how ethical (propriety) someone is. appreciation resources are concerned with feelings about entities or natural phenomena. It can be divided into ‘reactions’ to things as engaging or uninviting, and evaluation of their ‘composition’ as balanced or discordant or their ‘value’ as profound or insignificant.

A given attitude can be realized across a range of grammatical categories. Typical inscribed realizations are adjectives (e.g., happy), adverbs (e.g., happily), and verbs (e.g., enjoy). Furthermore, ideational meanings can be selected to invoke these meanings. For example, “He is always trying to learn” is an indirect invocation of tenacity by alluding to determination rather than using explicit attitudinal lexis (e.g., hardworking). To fully describe evaluative meaning in a text, we take into account the emoter (i.e., the conscious participant who experiences the emotion) and the trigger (i.e., the phenomenon responsible for that emotion) when analyzing affect, and consider the appraiser (i.e., the source of the attitude) and the appraised (i.e., what is being appraised) when analyzing judgement and appreciation [19]. For more delicate types, we should also be concerned with the [high-low] cline of intensity (e.g., extremely vs. somewhat or ecstatic vs. happy) and oppositions, such as [positive] vs. [negative] (e.g., happy vs. unhappy), [surge] vs. [disposition] (e.g., cry vs. upset), etc. [24,25].

We begin our analysis with a close reading of the entire dataset, and we code the texts for evaluative instances according to the appraisal framework. Employing a binary gender perspective, we compare patterns of evaluation targeted at men and women in each category. Based on the differing attitudinal representations of each gender, we then interpret how gender relations are reflected through evaluative language in Chinese news reports.

## 3. Results

### 3.1. Overview of Data Findings

We notice that ‘positive’ valence is prominent across all three kinds of attitudes, characterizing both the headlines and main themes of most of the news coverage. Instances of positive judgement towards people’s character are further enhanced by sharp contrast to the adverse situations healthcare workers encounter, which invoke a sense of ‘insecurity’, including excessive workload and unpleasant working conditions. In regard to appreciation, the focus is on objects and phenomena instead of people. Instances of affect deal with the strong feelings of women when they become the emoter.

### 3.2. Patterns of judgement

#### 3.2.1. Patterns of capacity

A salient pattern encountered in the analysis is that news reports prefer to select ideational meanings (i.e., experiential reality, such as someone has done something) to invoke the judgement of capacity, rather than using attitudinal lexis (as realized by adjectives and adverbs, such as ‘competent’ and ‘brilliant’) that tells us directly how to feel. These news reports are always result-driven to show what kinds of tasks have been completed and the outcomes that have been achieved. For example:

(1) The affiliated hospitals of the Tongji Medical College of Huazhong University of Science and Technology have devoted more than 33,000 medical staff and 8900 hospital beds, and two renowned affiliated hospitals of Wuhan University have devoted more than 17,000 medical staff and 8600 hospital beds. (CCTV news, http://bitly.ws/A4tS, (accessed on 28 November 2022)).
(2) The eighth batch of medical team from Chongqing to aid Hubei consists of 163 medical staff. It received 83 patients and 67 of them were cured… It’s the “first” one to deploy the ECMO technique, prescribe traditional Chinese medicine apozem, and conduct psychological intervention and respiratory rehabilitation training. (*Chongqing Daily*, http://bitly.ws/A4zq, (accessed on 28 November 2022)).
(3) In Wuhan, 3047 League members specialized in healthcare displayed courage when placed in harm’s way and held fast to their positions in fever clinics, observation rooms, emergency rooms, intensive care units, and grassroots communities; 2000 Youth League members from 97 commandos in Jiangsu Province provided help for 58 manufacturers and produced more than 1076 masks and 130,000 protective suits; in Xinxiang of Henan Province, eight youth commandos helped 15 key enterprises to produce 1,100,000 masks per day; and in Changxing, Zhejiang Province, 68 League branch secretaries took charge of croplands and aided in the reaping, packaging and delivering of farm products. (*China Youth Daily*, http://bitly.ws/A4Ez, (accessed on 28 November 2022)).
(4) Among the 42,000 medical staff that have been sent to Hubei Province to fight against the pandemic, 12,000 were Post-90s (born in the 1990s), among which Post-95s (born between 1995 to 1999) and Post-00s (born in the 2000s) accounted for a substantial portion. (China Youth Net, http://bitly.ws/AtVG, (accessed on 28 November 2022)).

In the examples, hospitals show their strength in providing necessary resources, such as personnel, fields, and devices (example 1). People make contributions to defeating the virus (examples 2–4). Yet, reports are preoccupied with numerical expressions regarding the medical staff present (example 1), patients treated or discharged (example 2), and young people’s dedication on behalf of their League, provinces, or generations (examples 3–4). Within the large groups of hospitals and medical teams, there are subdivisions categorized by specific medical performances, such as:

“Blood purification squad, which was made up of 7 people”;

“Xia and Meng’s team of the Anesthesiology Department of the East Zone”.

Similarly, in these examples, we only get information about how many members there are and their leaders. As a result, women as individuals tend to be concealed by the group orientation.

However, the collectivist orientation of Chinese media does not seem to conceal the contributions of male individuals. The reason is that men are reported as the core of the groups due to the overrepresentation of their expertise in therapy and management. In our data, among the ordinary healthcare workers that can be identified by gender, men are medical technicians in charge of various departments (mentioned 20 times), heads of hospitals (19 times), heads of medical teams (11 times), nurses (five times), and so forth. In contrast, nursing roles account for the majority of female workers (mentioned 53 times), followed by logistic and liaison workers (three times), team heads (three times), obstetricians (three times), directors of departments (twice), and one from the infection-control department. In an episode of a news channel, among the six spokespersons shown in the videoclip, the vice principal, the administrative manager, and the general secretary of universities or hospitals are all males. When a male general secretary of the hospital pays tribute to those who carry out dangerous work, such as performing endotracheal intubation in patients with respiratory failure in intensive care units, female workers are backgrounded most of the time. Females are in a large crowd, dressing up in protective suits and going unrecognized in the so-called “dare-to-die intubation team”. (CCTV news, http://bitly.ws/A4tS (accessed on 28 November 2022)) That is a typical representation of women in our video data.

Below are more examples reporting the on-site visits to Wuhan (Bold: identity):

(1) **Mr. Peng**, **director** of the intensive care unit of Zhongnan Hospital, says ECMO is only for rescue purposes and is not suitable for popularization. This technology is a life support for critically ill patients with cardiopulmonary failure… **Head nurse Ms. Ma** says beds and protective suits are in short supply. She asks nurses to avoid drinking too much water and reduce the chances of taking the suits off… (*China Newsweek*, http://bitly.ws/A57R, (accessed on 28 November 2022)).
(2) **Mr. Deng**, **director** of the vascular surgery department, remembers the terrifying surgery. Sight and movement were largely restricted due to foggy goggles and the protective suit, but he successfully separated the femoral vein and the femoral artery thanks to extensive experience. (*China Youth Daily*, http://bitly.ws/A57N, (accessed on 28 November 2022)).
(3) **Mr. Gong**, **head** of the medical teams of Dalian Province, remembers that the degree of blood oxygen saturation of a most critical patient was only 39%, in a severe hypoxic condition… **Nurses** take the hardest work as they keep giving injections, drawing blood, carrying out adjuvant therapy and assisting patients eating, going to the toilet, and disposing of waste. (China National Radio News, http://bitly.ws/A57J, (accessed on 28 November 2022)).
(4) Makeshift hospitals (“Fangcang”) were born following the suggestion of **Mr. Wang**. He is the **dean** of the Chinese Academy of Medical Sciences and vice dean of the Chinese Academy of Engineering. … **Mr. Wan**, **vice dean** of Hubei General Hospital and dean of the makeshift hospital in Wuchang, guided his team in drawing up the work manual for the makeshift hospitals, which was then adopted by the headquarters and issued to each branch of the makeshift hospitals. (*Guangming Daily*, http://bitly.ws/A57G, (accessed on 28 November 2022)).
(5) After the advice of the **team chiefs**, **Mr. Chen** and **Mr. Hu**, a series of key measures were implemented, such as quarantine, disinfection, fever clinic and pre-examination triage. … in February, **Mr. Hu** led other medical workers in the department and was stationed in the East Area. (*The Paper*, http://bitly.ws/A57E, (accessed on 28 November 2022)).

The examples show that males’ discourse is more laden with technical words, such as example (1) introduces the usage of ECMO (extracorporeal membrane oxygenation), example (2) concerns the surgery process, and example (3) deals with the condition of a critical patient. Meanwhile, it is more possible for men to be leaders of party and government offices, especially in terms of their dominant decision-making power during the pandemic in advising the establishment of makeshift hospitals, writing work manuals, and implementing key measures (examples 4–5). However, the issues that women deal with require fewer professional skills, as their usual work often includes performing intubation, transfusion, and suctioning and recording patients’ conditions, as well as being housekeepers who manage care and deliver meals for patients. As a result, men seem to be a major force and play a more significant role than women in improving the pandemic situation.

The judgement of capacity is positioned to unite readers as appraisers for those who have exploited their expertise to control the virus. However, the persons being appraised are always group entities, including hospitals, teams, and generations, and there is a higher possibility of men being representatives of these groups. As a result, only a small number of women are representative of these groups. This limits the inclusion of women as protagonists in Chinese coronavirus news coverage.

#### 3.2.2. Patterns of tenacity

Compared to the judgement of capacity, the qualities of tenacity and propriety favor exclusive stories, which offer in-depth and extensive portrayals of specific female workers. The stories were recounted by personnel, themselves involved in routine work from the start of the Wuhan rescue until its conclusion, and included personal anecdotes from the wards and feelings from the perspective of healthcare workers. These stories help to create fuller images and multifaceted identities of female workers.

tenacity means people are plucky, brave, heroic, reliable, persevering, or resolute [24]. Journalists deploy lots of linguistic resources to portray this quality. Repetition, either by repeating the same lexical item or assembling lists of terms closely related semantically, is one of the ways to intensify meanings [19]. In our data, this mode of intensification is mainly realized by Chinese four-character idioms or parallel sentences. Some examples are translated as follows:

Ms. Zhu led her team in monitoring hospital infection unceasingly and relentlessly.

Ms. Du, though has a slight figure, is strong-willed, independent, and accountable.

The heroines stepped forward bravely, and were not frightened of doing so.

They were assiduous and willing to devote, persevering without rest, and when the going gets tough, the tough get going.

However, some reports compare how women act today with how they used to be, suggesting women as the weaker sex (examples 1–4), while men are inherently described as stronger and braver (example 5) (Bold: identity):

(1) they (**women**) take off usual dresses and put on wartime robes (xinhuanet, http://bitly.ws/A6i9, (accessed on 28 November 2022)).
(2) The tender shoulders of **women** can carry a heavy load. They are as excellent as their male peers…

Though **women** are tender and weak in stature, they stand fast as if they’re huge rocks…

They’ve founded a fortress with both delicate and strong qualities. (ibid.)

(3) **Women** are inherently weak, but they become stronger when fighting against the pandemic. (Xinhua News Agency, http://bitly.ws/A6ia, (accessed on 28 November 2022)).
(4) **Ms. Huang**, like many other nursing sisters in the rescue, had kinfolk to look after her. But in Wuhan, she becomes a warrior. (*People’s Daily*, http://bitly.ws/A6ic, (accessed on 28 November 2022)).
(5) What he wrote in the battle request is …young generation should be vigorous, and **men** should have the courage… (http://bitly.ws/A6ie, (accessed on 28 November 2022)).

We can see that ‘weak’ has become a keyword to describe women. Example (1) is adapted from the Ballad of Mulan [26]:

“I take off my wartime gown

And put on my old-time clothes.”

Facing the window she fixes her cloudlike hair,

Hanging up a mirror she dabs on yellow flower-power.

This is a legendary folk heroine who took her aged father’s place in the army by disguising herself as a man. The two verses talk about the scenario where she retires to her hometown and needs to change her clothing. Example (1) in our data imitates the way she dresses up as a warrior. Considering that women’s attire is stereotyped as more concerned with aesthetics (example 1), they’re not as capable as male peers when dealing with heavy loads (example 2) and need to be taken good care of (example 4); we can hardly associate ‘war’ with women. Yet, women move beyond the gender barrier and choose to be as heroic as Mulan in combating the virus. The implication is that no one has a reason not to stand up, as the weak have already prepared to do so [27], which would be a most effective mobilization of the public.

In addition to attitudinal words, the unpleasant working conditions on the frontline also intensify the sense of tenacity to a larger extent. Both female and male health workers overcome work overload, highly stressful work racing against time and death, and physical exhaustion. However, nuanced differences exist in regard to what type of work tests them. For example (Bold: identity):

(1) In the early days of the outbreak of COVID-19 in Wuhan, daily outpatient visits were 10 times those of the past. **Ms. Zhu**, **a nurse** from the nursing team of the fever clinic, worked from 2 a.m. to 9 a.m. and did blood sampling for over 200 patients. She sweated in her protective suit profusely but could not take off her gear for long periods of time. Her face was red and swollen afterwards… (*Economic Daily*, http://bitly.ws/A4Ae, (accessed on 28 November 2022)).
(2) **Ms. Huang**, who took charge of 3 neighborhoods that consisted of 1831 households as well as 5 hotels, 2 construction sites, and 1 supermarket, had to walk 20,000 steps every day. (CCTV News, http://bitly.ws/A6j6, (accessed on 28 November 2022)).
(3) **Mr. Yu**, **the head of the 21st ward**, has worked continuously for 40 days and saved more than 60 critically ill patients with no staff being infected. Even without the help of X-ray radiography machine, he managed to set up the temporary cardiac pacemaker for patients. … **Ms. Guo**, **a nurse** from the emergency centre, carried out health care work for more than 10 h a day… helping patients breathe, drink water, have meals, go to the toilet and change their positions in bed…everything worked out, but she was infected unfortunately… She volunteered to go back to the frontline once she recovered from the COVID. (*The Paper*, http://bitly.ws/A6jq, (accessed on 28 November 2022)).
(4) The first three days at ‘Fangcang’ were the hardest. **Chief Mr. Chang** and his teammates work day and night to work out the schemes of treatment methods, diagnostic standards and discharge standards… (*Guangming Daily*, http://bitly.ws/A57G, (accessed on 28 November 2022)).

The examples show that the work of women on the frontline seems to be less demanding than that of men. Reports focus on women’s nursing work (examples 1 and 3) and non-healthcare occupations (example 2), with repetitive heavy work requiring long hours. However, the test for men is more than physical strain; it pertains to skills and abilities, such as surgical proficiency, decision making, and command. In examples 3 and 4, men overcome challenging situations when key equipment is not available, and they get through difficult times at the initial stage by thinking up strategic policies and plans.

#### 3.2.3. Patterns of propriety

propriety is connected to good, moral, ethical, law-abiding, kind, or caring evaluations [24]. In our data, frontline workers’ practices are associated with social values, such as selfless dedication, care for others, and civic duty. These opinions about people and the way they behave contribute to the standard of good or bad and right or wrong in China for social living as a citizen, a doctor, a mother, or even a woman. Ultimately, they shape the social ideal of how women should behave.

Both male and female workers on the frontline make personal sacrifices by giving up safe working conditions, free time, and even their lives. For example, the tracheotomy in COVID-19-positive patients could be a high-risk procedure for medical personnel, but a doctor says:

“…as doctor and party member, it’s duty-bound to do so as long as patients can benefit from it” (*Guangming Daily*, https://epaper.gmw.cn/gmrb/html/2020-03/30/nw.D110000gmrb_20200330_1-09.htm (accessed on 28 November 2022)).

There are more vivid descriptions to show people’s self-sacrifice for the sake of others. One way involved using maximisers to upscale meanings that construe upscaling as being at the highest possible intensity [19]. For instance,

Ms. Zhu braved into the most critical and sensitive parts to make sure everything was in place.

The head nurse Ms. Wang always rushed at the foremost to do the hardest jobs.

Moreover, invoked evaluation by ideational meanings allows readers, especially those who are sympathetic to the experiences in the text, to align with the values naturalized by the text [19], e.g.,

They ate and slept in the hospital, never went home, and never took a rest for a single day.

They worked hard and even forgot to eat food.

They kept working till the small hours.

However, reports fail to notice the anatomical and physiological sex differences that determine the limits of human performance [28]. For instance, due to a different circadian timing system [29], women have greater sleep maintenance problems and are predisposed to insomnia; as the basal metabolic rate varies significantly with the menstrual cycle [30], women easily become fatigued during menstruation; women tend to suffer more from mental illness, as some studies point out females’ psychological state after the outbreak of COVID-19 in Wuhan [31,32,33,34]; and women may have higher rates of physiological maladies because of the shortage of fresh air in unventilated, makeshift compartments while wearing airtight protective suits. On the contrary, Chinese media are keen on shaping these female characters as moral exemplars for public emulation. For example:

(1) **Ms. Cai**, **a nurse** from the Hankou Hospital, who just recovers from the COVID, is now donating plasma. She will be back to work at hospital on 22, February. … **Ms. Guo** works 10 h per day voluntarily and it’s often the case to work around the hour… **Ms. Song** sleeps less than 4 h per day. She works in “perpetual motion” including venipuncture, intubation, sampling, feeding patients and having chitchat with old patients. (China National Radio News, http://bitly.ws/A6aY, (accessed on 28 November 2022)).
(2) **Mrs. Zhang** has been pregnant for 5 weeks. The director finds her continuing the night shifts. (China Youth Net, http://bitly.ws/A6cc, (accessed on 28 November 2022)).

These female workers get over physiological barriers and carry out all kinds of work (example 1), even if they’re pregnant (example 2).

The sense of sacrifice is magnified when women, whose primary identity is motherhood in societal gender expectations [35], have to be apart from their children. News reports like to describe women’s sacrifice as they prioritize combating the coronavirus for the nation rather than taking care of their families. According to our data, the co-occurrence of women with family is three times more than men (21 vs. 7), and the ratio with children alone is higher (15 vs. 2). When men co-occur with children, there is a high possibility that women are by their side as wives. For example (Bold: identity):

(1) **Mr. Shen**, a doctor from urinary surgery to rescue the fever clinic, says his wife warns him to wash hands regularly and his son wants to be a “Dragon Force warrior” to fight with him. (*Guangming Daily*, http://bitly.ws/A6cJ, (accessed on 28 November 2022)).
(3) **She** was the first one to apply for the fight after the notice of volunteer recruitment. At that time, her youngest son was only 10 months old. (xinhuanet, http://bitly.ws/A6dr, (accessed on 28 November 2022)).
(4) Even if her daughter was at a crucial period for the entrance examination, **she** left her to the elderly and took one for the team. (*Economic Daily*, http://bitly.ws/A6ds, (accessed on 28 November 2022)).
(5) **Mrs. Zhou**, a medical member from Anhui Province working at the makeshift hospital of Wuhan Sports Centre, left a message for her son on the protective suit “Chen** from the Hefei No.45 High School, please study hard!” (China Internet Information Center, http://bitly.ws/A6dt, (accessed on 28 November 2022)).

The examples show the main burden of childcare falls on women when children are infants and have schoolwork to do. Both require substantial amounts of time and attention, and women are more likely than men to play the role of primary carer to children, which society expects of them.

In addition to showing sacrifice and unwavering devotion to the state’s project of combating the pandemic, feminine qualities stand out in these stories. In cases where babies were separated with their family members in the isolation wards, female nurses took on duties to take care of their diets and daily lives. These female healthcare workers were called “temporary mothers” (People’s Daily Online, http://bitly.ws/Ag3Q, (accessed on 28 November 2022)) and “superhuman mothers” (People’s Daily Online, http://bitly.ws/Ag3E, (accessed on 28 November 2022)). These titles suggest that women are placed at the heart of nursing and childcare obligations. One more example:

Several nurses worked in shifts to feed and bathe the baby ‘Lele’, change nappies, and tuck him in. These ‘moms’ have tried different ways to play with him and sometimes draw pictures to catch his attention. Once he cried after awaking, the tender ‘moms’ would come to lull him… he stayed in the nurses’ arms for almost half of the day. (*People’s Daily*, http://bitly.ws/A6k7, (accessed on 28 November 2022)).

The detailed descriptions of feminine maternity provide hints about what is ‘proper mothering’ or what is considered a ‘proper woman’. Even though the mother–child bond is beyond the ties of kinship, these mothers hold the family infrastructure together by their virtues of caring, ethics, teaching, and service, and move toward the mutuality of a shared sense of sisterhood that binds women as a community [36].

The illustrations above regarding how ethical someone can be include a full range of social dimensions that women possess, from professional to domestic identities. However, their representation in news reports center around sacrifice, dutifulness, and care, which could be highly demanding, as sometimes these situations may run counter to the everyday situation.

### 3.3. Patterns of appreciation

As complementary to the evaluation of people and their behaviors, appreciation resources are concerned with aesthetic evaluations about entities and phenomena. In our data, the stage when medical workers sent to Wuhan return home is characterized by gratitude and appreciation from citizens or the government, with an assessment of the social significance of the process of combating the pandemic (+valuation). There are also positive representations of the configuration of makeshifts wards, which accommodate various complex and diverse functions (+composition).

However, the gendered narrative is prominent regarding the appreciation subcategory ‘reaction’, which is about the emotional impact that products and performances have on us, such as ‘captivating’, ‘lovely’, ‘beautiful’, ‘appealing’, ‘dull’, or ‘unremarkable’. For this dimension, we can hardly find a male match for comparison. Physical attributes become the most pronounced part of the stereotype, according to four gendered features proposed by Deaux and Lewis [37]—qualities, role behaviors, jobs, and physical characteristics.

In our data, the body narrative favors women over men, either in words or images. It is common to see news outlets choose women as news covers: staring female health workers with tearful eyes, worried looks, and sweaty faces. This supports Jia et al.’s findings [38], based on a large-scale and data-driven study, that media are more likely to represent women visually when they were mentioned as a news actor or source.

Special reports reached a peak on International Women’s Day, and their headlines are:

“You, are the most gorgeous roses combating the coronavirus in this spring”.

“They [a word with feminine gender in Chinese] being the most charming people”.

“Paying tribute to the most adorable ‘She’”.

“The masks cannot hide your beauty: to the frontline”.

In the body paragraphs, women are named as the “prettiest good catches”, and “scars on faces” were compared to the makeup of COVID.

In addition to facial features, women’s hair has also attracted a lot of attention. Female workers cutting their beloved hair became a hot topic. A bareheaded nurse has become a trending news story for a long period of time. Examples are:

(1) **Ms. Luo** cried for those sisters who are in an age where great efforts are made to achieve beauty, but they have to cut their long hairs for the sake of efficiency in the wards. (Xinhua News Agency, http://bitly.ws/A6kx, (accessed on 28 November 2022))
(2) For the sake of work and safety, **Ms. Wang** cut her beloved hair. Upon seeing her picture, her mom cried, “she never wore short hair before. Her smooth and attractive hair is her ‘lifeblood’. I’m so moved by her courage.” (*The Paper*, http://bitly.ws/A6kz, (accessed on 28 November 2022))
(3) The ‘**bald angel**’ cut ‘Qing Si’. (Xinhua News Agency, http://bitly.ws/A6kB, (accessed on 28 November 2022))

There is an underlying stereotype that men have short hair, while women have long hair in order to fit their clothing and accessories. Women’s hair even has special meanings in ancient China, as shown in example (3). It is called ‘Qing Si’ (an ancient phrase meaning the black hair of a woman), which is regarded as a symbol of love and can be a woman’s love token when getting married. Moreover, ‘Si’ (the shape of hair) has the same sound as ‘Si’ (missing someone) in Chinese, and women will give a pinch of hair to their love in farewells. Surprised reactions to hair cutting derive from female workers failing to correspond to stereotypical female qualities and engaging in behavior that may lessen their beauty.

### 3.4. Patterns of affect

In this section we turn to women as conscious participants experiencing emotion. In this research, it was discovered that male and female figures differ a lot in emotional patterns regarding the kinds of feelings and targets, and the triggers of feelings in narratives.

Women are characterized as more sentimental, with their feelings being realized as a surge of emotion. This is manifested extra-linguistically through behaviors such as “cry” and “tears” (examples 1–3), whereas the predisposition of males is rational and undemonstrative, with more internal emotional experiences (example 4) (Bold: identity):

(1) **Ms. Huang** sobbed for the enthusiasm and warmth from Wuhan citizens. She said she has never been so moved. When she saw the bus driver, who was of the same age as her father and was in charge of the shuttle between the hotel and makeshift hospital, eat snacks right beside the flowerbed outside the hotel, she was bitter beyond expression. (Xinhua News Agency, http://www.xinhuanet.com/politics/2020-03/27/c_1125776495.htm, (accessed on 28 November 2022)).
(2) **Ms. Zheng**, a shop volunteer, could not stop crying when receiving dozens of solicitudes from relatives and friends. (*The Paper*, https://www.thepaper.cn/newsDetail_forward_6690889, (accessed on 28 November 2022)).
(3) A patient **Ms. Fu** was moved to tears when medical staff celebrated her birthday. (*Economic Daily*, https://baijiahao.baidu.com/s?id=1662048282545234649&wfr=spider&for=pc, (accessed on 28 November 2022)).
(4) **Mr. Gong** remembered that after finishing 5 surgeries for critically ill patients, he heard that there were 8 more to come. He immediately felt worried and stressed for the imminent fight. (China National Radio News, http://dl.cnr.cn/dlrw/20200329/t20200329_525034171.shtml, (accessed on 28 November 2022)).

In the examples, women have greater tear-eliciting emotions, while men’s feelings are presented by descriptions. These examples also provide information about the context in which the emotions occur. Female crying is triggered by exemplary deeds, such as the willingness to make significant personal sacrifices, and being generous, helpful, or considerate, while the emotional experience of a man is related to the pressure of social responsibility. Although a lot of empirical research on sex differences in emotions indicates that men do cry and that this can be perceived positively [39,40,41], our data tend to detail the emotional expressions of women workers or patients rather than men. Men’s emotional inexpressiveness clearly demarcates them from females and femininity.

Bodenhausen and Richeson [42] point out that stereotypes not only represent the features ascribed to social groups, but also involve causal chains that relate group characteristics to one another. The causality invoked in the affect patterns is that men are hardly caught crying because “their attributed traits include being brave, fearless and tough while women are supposedly weak, fearful and fragile” [43]. The group’s inherent “essence” thus constitutes a coherent account for why the group is the way it is perceived to be. The concealed displays of emotion of men are emotionally appropriate to show their higher level of tolerance and reliability. By contrast, female characters in the news become an effective outlet for crying associated with sadness, frustration, feelings of overwhelm, and ‘being touched’. As crying prompts others to pay attention and is a powerful form of persuasion [41], the exemplary acts that trigger crying are a means to connect with the presumed target audience and general public.

## 4. Discussion

To summarize the findings above, we find that uniting readers as an appraiser to judge the capacity, tenacity, and propriety of frontline teams or workers is the main theme of the Chinese COVID-19 frontline reports. The attitudinal valence is constantly positive. As journalists strategically adopt shared feelings in the face of risk events [44], the positive attitudes in Chinese narratives serve as the means to negotiate solidarity with those who are from the same cultural backgrounds and are sympathetic to the experiences in the text. This alignment helps build up confidence in combating COVID-19. Yet, after close investigation of the persons being appraised, we argue that Chinese newspapers’ narrative of women needs to be improved regarding the underrepresentation of their contributions, moral burden, and marginalized professional identity. Patterns of three subcategories of judgement show that journalists emphasize two relating dimensions more in the presence of women, i.e.,tenacity and propriety, rather than capacity. The two categories are more concerned with people’s characters and are closer to moral judgements of praise or condemnation, whereas women’s professional abilities as experts or workers are underestimated. Meanwhile, the aesthetic appreciation of women and the way women express feelings, as well as the ethical standards of proper mothering or the ethical standards of women, reveal the underlying gender bias infused by journalists.

Global statistics show that women only represent 25% of subjects and sources in the news, with no significant incremental change during the last monitoring period [45]. China’s inclusion of women as protagonists in coronavirus news coverage conforms with this pattern. Our findings suggest Chinese media focus on team names more than individuals due to their goal orientation and group interests. In these groups or teams, men’s voices are predominant, both in the COVID-19 treatment and the hospital management. They play the leading role as decision makers in the administrative domain and have core professional and scientific expertise in improving the pandemic situation. Ross [46] points out that who is asked to comment on and in the news is a significant indicator of who “counts” in society and whose opinions have legitimacy and status. In Chinese COVID-19 frontline reports, however, women do not have an equal opportunity to be representatives of a team, not to mention having the advantage of standing out to show their contributions of knowledge and expertise.

News discourse is a kind of genre, i.e., “a staged, goal-oriented social process” [24]. The COVID-19 frontline reports of China’s mainstream media are ideologically committed in the service of dominant Chinese cultural values. They set the standards for what is a good citizen, a good doctor, or a good woman. Female frontline workers are labeled as Jinguo Yingxiong (heroines), contemporary “Mulan” (a legendary folk heroine in Chinese history), “roses combating the COVID”, and Goddess. The articles assert that “women hold up half the sky”. They brave long stays, exhaustion, overtime, and additional working, just as men do. Yet, in fact, they hold a heavier burden than men because physiological differences predispose them to insomnia, psychological problems, fatigue, and physiological maladies. However, news reporting tends to shape them as transcendent heroes who break through sex barriers and have done even more than men. As external influences push people to comply with standards in society [47], female workers on the frontline have to conform to social expectations to uplift spirits and morality. This places considerable pressure on everyday women, however.

Last but not least, gender stereotypes still exist in female narrations. Journalists pay a lot of attention to the feminine and maternal attributes of female frontline workers, such as attractiveness, patience, considerateness, and connection with family issues. However, this may reinforce a gendered division of labor that distributes men and women differently into social roles in the home and at work [48]. As a result, it distracts people from women’s professional identity. Hentschel et al. [48] finds that in the workplace, the predominant facets of female workers are concern for others, sociability, and emotional sensitivity, signifying they are typically rated higher on ‘communion’ but lower on ‘agency’. ‘Communion’ means being attuned to others and building relationships, while ‘agency’ is to take charge and be in control. In our data, feminine qualities stand out in the stories. They comforted and attended to patients patiently and carefully, paid careful attention to every detail, and offered warm-hearted help when taking part in healthcare work. In contrast, males show more assertiveness and leadership competence. This also coincides with Lippa et al.’s finding [49] that women have tended to be employed in people-oriented, service occupations rather than things-oriented, competitive occupations that have traditionally been occupied by men.

At the same time, Hentschel et al. [48] also finds that women’s close connection with children prompts an inference that in the domestic sphere, women have performed the majority of routine domestic work and played the major caretaker role. Even in medical workplace scenarios, they face societal expectations regarding family roles, especially attributes of ‘motherhood’. “Mothers are not born; they are made” [50]. The high rate of women being photographed and associated with feminine attributes and domestic duties reinforces the values and expectations of the dominant culture. Motherhood becomes a male-controlled and deeply oppressive patriarchal institution that defines women’s own experiences of mothering [51].

In China, however, the engraving of sacrifice and devotion in people’s minds may result in no one turning down or doubting gendered situations during COVID-19. There are external pressures exerted from the socio-cultural values. They encourage people to do what is expected as good and to avoid what is considered as inappropriate. Social morality in China tends to be regarded as conducive to maintaining public interests and social order and contributing to the advancement of socialism. Some of the gendered situations relate to morality and may be tolerated and accepted by the public. For example, women’s open displays of emotions always respond to people’s exemplary acts so as to positively evaluate people’s character; the feminine and maternal attributes regarding the caring facet and sense of community beyond kinship are indispensable parts of socialism; and the character design of weaker women can also be a useful mobilization of the public to make contributions.

## 5. Conclusions

Gender issues during the COVID-19 pandemic have been a focus of discussion around the world. This paper has investigated gender relations in China by deploying an appraisal-based approach to analyze a dataset consisting of news about the COVID-19 frontline from Chinese mainstream media. Media representations of women in relation to occupation and family during the COVID-19 crisis were exemplified in detail according to the patterns of judgement, appreciation, and affect. The research indicates that the positive judgements of handling tough work, dedication, caring for others, and so on help build shared feelings towards the community and reconstruct the disturbed social order. However, some space remains for the improvement of gender relations in China. The discussion section further explored women’s overshadowed career ability, the moral burden placed on them, and journalists’ gender bias when focusing on women’s physical attractiveness and nursing or mothering qualities.

This paper thus offers insight into the investigation of gender equality in media discourse, as well as gender relations in China during the pandemic. However, there are still important issues around this topic that need to be resolved. For example, the semantic domain of the linguistic expression of emotion can be enriched by combining linguistics with cognition and psychology. These disciplines have more detailed classification of the kind and amount of expressions and would help explain the nature of various emotions. Second, evaluation is linguistically expressed by an open-ended set of forms, and the appraisal framework is a “flexible interpretative tool” [52]. Though we have described the design of a clear coding scheme by conducting pilot studies and identifying potential problems, it is still likely that some subjectivity remains in the identification of attitudes. The last point concerns the technical issues of the appraisal system: when applying the framework to texts, hybrid realizations are often encountered. This means judgement of character, appreciation of things, and the surge of affect can occur simultaneously. Sometimes, the positive or negative appreciation of things equates an evaluation of their actors when an emotional reaction is also enacted. Our study has not paid much attention to the recontextualization of different categories of attitudes. Future research may seek to investigate the hybrid, as well as implicit, evaluations that rely more on pragmatic and contextual implications.

## Figures and Tables

**Table 1:** List of news agencies and numbers.

**Media Name**	**Introduction**	**Number**
***People’s Daily*** (People’s Daily Online/ People’s Forum/ cpcnews.cn)	The largest newspaper group in China that provides direct information on the policies and viewpoints of China. http://www.people.com.cn/, http://www.rmlt.com.cn/, http://cpc.people.com.cn	26
Xinhua News Agency (Xinhuanet/ ***Xinhua Daily Telegraph***/ xhby.net)	The official state news agency of China, and the largest news- and information-gathering and release center in China. http://xinhuanet.com/, https://www.xhby.net	21
***Guangming Daily*** (Guangming Online)	Popular among Chinese intellectual circles, and focuses on the fields of education, science and technology, and culture and theory. https://www.gmw.cn	18
***The Paper***	A Chinese digital newspaper owned and run by the Shanghai United Media Group. https://www.thepaper.cn	16
China Internet Information Center	A key state news website for China in international communication. It provides round-the-clock news services in 10 languages. http://www.china.com.cn	12
CCTV News (cctv.com)	A news channel of China Central Television. https://news.cctv.com	10
***Health News***	An authoritative national newspaper on the healthcare industry run by the National Health Commission of China. https://www.jkb.com.cn	6
***Worker’s Daily***	The official newspaper of the All-China Federation of Trade Unions. https://www.workercn.cn	6
China National Radio News	A national radio network of China owned by the state-owned China Media Group. https://www.cnr.cn	5
***China Youth Daily***	An official newspaper of the Communist Youth League of China, whose target audience is young people. http://zqb.cyol.com	4
***Economic Daily***	A Chinese newspaper focusing on economic reports. http://paper.ce.cn/, http://www.ce.cn	3
***Jiefang Daily***	The official daily newspaper of the Shanghai Committee of the CCP. https://www.jfdaily.com	2
***People’s Liberation Army Daily***	A newspaper covering news stories relating to the PLA and other military affairs. http://www.81.cn	2
China News Service/ (***China Newsweek***)	The second largest state news agency in China, directed at overseas Chinese diaspora worldwide. (*China Newsweek* is a Chinese weekly magazine that covers domestic and international news, specializing in current affairs, culture, and politics.) https://www.chinanews.com/, http://www.inewsweek.cn	2
China Youth Net	The largest mainstream media platform for young people in China. https://www.youth.cn	2
***Sichuan Daily***	A leading Chinese language daily newspaper based in Chengdu, Sichuan Province. https://www.scdaily.cn	2
***Qiushi***	A leading Chinese theoretical journal whose contributors include scholars and researchers of China’s think tanks and academic institutions. http://www.qstheory.cn	2
***Science and Technology Daily***	The official newspaper of the Chinese Ministry of Science and Technology, regarded as the authority for science and technology issues with objective and scientific perspectives. http://digitalpaper.stdaily.com	2
***Hebei Daily***	A leading Chinese language daily newspaper based in Shijiazhuang, Hebei Province. https://hbrb.hebnews.cn	2
ScienceNet	Supported by the Chinese Academy of Sciences, the Chinese Academy of Engineering, and the National Natural Science Foundation of China, with the mission of establishing a global Chinese science community. http://www.sciencenet.cn	1
***Guangxi Daily***	A leading Chinese language daily newspaper based in Guangxi Province. https://gxrb.gxrb.com.cn	1
***Shaanxi Daily***	A leading Chinese language daily newspaper based in Shaanxi Province. https://esb.sxdaily.com.cn	1
***Chongqing Daily***	A leading Chinese language daily newspaper based in Chongqing Province. https://www.cqrb.cn	1
***China Education News***	A national newspaper focusing on the field of education in China. http://paper.jyb.cn/	1
***Beijing Daily***	A leading Chinese language daily newspaper based in Beijing Province. https://www.bjd.com.cn	1
***Hubei Daily***	A leading Chinese language daily newspaper based in Hubei Province. https://epaper.hubeidaily.net	1

## Data Availability

Publicly available datasets were analyzed in this study. This data can be found here: https://www.whu.edu.cn/xxfy/xwgz.htm, (accessed on 28 November 2022).
